# Reactivation of p53 via MDM2 inhibition

**DOI:** 10.1038/cddis.2015.302

**Published:** 2015-10-22

**Authors:** E S Kim, J M Shohet

**Affiliations:** 1 Division of Pediatric Surgery, Department of Surgery, Keck School of Medicine, University of Southern California, Los Angeles, CA 90027, USA; 2 Department of Pediatrics, Section of Hematology–Oncology, Texas Children's Cancer Center, and Center for Cell and Gene Therapy, Baylor College of Medicine, Houston, TX 77030, USA

At diagnosis, >98% of neuroblastoma are p53 wild-type with intact downstream apoptotic machinery.^1^ In addition, even after multiple rounds of dose-intensive chemotherapy, a large majority of recurrent neuroblastomas maintain p53 functionality.^1^ This suggests that for neuroblastoma patients with both *de novo* and relapsed conditions, reactivation of p53 and induction of p53-dependent apoptosis may be an effective therapeutic strategy for the majority of *de novo* and recurrent neuroblastoma tumors.^1^ In a recent issue of *Cell Death Discovery*, Lakoma *et al.*^2^ demonstrate potent antitumor effects for the novel MDM2 inhibitor RG7388 in multiple neuroblastoma tumor models.

As the major negative regulator of p53, the oncogene MDM2 has been found to be overexpressed in neuroblastoma and other solid tumor malignancies.^3^ MDM2 dramatically shortens the protein half-life of p53 by initiating ubiquitination via its E3 ligase activity. MDM2 inhibitors, which block the specific interaction between MDM2 and p53, can thus generate high levels of intracellular p53 and trigger apoptosis (Figure 1).^4^ The first specific and potent small molecule inhibitor to MDM2, Nutlin-3a, was developed by Vassilev *et al.*^4^ in 2004. Despite potent efficacy *in vitro* and *in vivo* in preclinical studies in multiple p53 wild-type malignancies, Nutlin-3a was found to have poor bioavailability, and as such, new generation MDM2 small molecule inhibitors were developed with the goals of improving potency, bioavalibility and pharmacologic properties.^5, 6, 7^

Initial efforts to optimize the Nutlin compounds led to the first investigational MDM2 inhibitor, RG7112.^6^ Preclinical evaluation of RG7112 demonstrated robust activation of the p53 pathway with a potent antitumor effect *in vitro* and *in vivo*.^8, 9^ Ray-Coquard *et al.*^10^ reported the use of RG7112 in 20 liposarcoma patients, and while many sustained gastrointestinal toxicity, the drug was found to activate the p53 pathway and demonstrate encouraging results. Further optimization of lead compounds led to the development of RG7388 (Hoffman-La Roche, Nutley, NJ, USA) that has improved bioavalability, potency and pharmacokinetics.^7^ Ding *et al.*^7^ found that RG7388 activates p53 at a significantly reduced concentration than RG7112, and this effect was seen both *in vitro* and *in vivo*.

In recent months, there have been several preclinical studies evaluating the efficacy of RG7388 in various cancers. Higgins *et al.*^11^ used a preclinical model of osteosarcoma to optimize dosing schedules for RG7388 and minimize toxicities seen with the daily administration of RG7112. They observed that weekly and biweekly dosing of RG7388 is as efficacious as daily dosing yet using lower doses of RG7388. Hai *et al.* tested the efficacy of RG7388 in several cell lines of non-small cell lung cancer.^12^ *In vitro*, they confirmed that RG7388 led to p53-dependent apoptosis of cancer cells, and *in vivo*, RG7388 significantly inhibited subcutaneous tumor xenografts from p53 wild-type cells but not tumors derived from p53 mutant cells. In addition in a rhabdomyosarcoma model, Phelps *et al.*^13^ found that RG7388 had very little single agent antitumor effect but provided synergistic antitumoral effect on xenografts when combined with ionizing radiation treatment. Chen *et al.*^14^ examined the effect of RG7388 in combination with chemotherapy in a panel of neuroblastoma cell lines. Using both p53 wild -type and p53 mutant cell lines of neuroblastoma, the authors found that RG7388 was most effective in killing p53 wild-type neuroblastoma cells, and this effect was synergistic with select chemotherapy agents. Lakoma *et al.*^2^ demonstrated that RG7388 effectively rescues p53 and activates downstream apoptotic pathways in p53 wild-type cell lines but not in p53 mutant cell lines in a number of *in vitro* assays. Using an orthotopic murine model of neuroblastoma, they also demonstrated that RG7388 potently inhibits neuroblastoma tumor growth in xenografts derived from several p53 wild-type cell lines, and that this inhibitory effect is abrogated in p53 mutated/null tumors *in vivo*.^2^ Immunostaining of the subsequent tumors confirmed increased tumor cell apoptosis in the RG7388 treated neuroblastoma xenografts from p53 wild-type cell lines, but not in the p53 mutated/null tumors.^2^

MDM2 inhibitors have also been found to activate broader, p53-independent pathways in cancer, which may contribute to their overall efficacy. The MDM2 inhibitor Serdemetan has been shown to have equivalent potency in both p53 wild-type and p53 mutant acute leukemia cells, the latter by E2F1-mediated apoptosis.^15^ In addition, Nutlin-3a was found to disrupt MDM2-p73 interaction and hence stabilize pro-apoptotic p73 in neuroblastoma, colon cancer and osteosarcoma cells.^16^ Nutlin-3a has also been found to competitively inhibit multi-drug resistant protein 1 and sensitize chemotherapy-resistant neuroblastoma and rhabdomyosarcoma cells.^17^ Patterson *et al.* previously demonstrated that MDM2 inhibition with Nutlin-3a suppresses hypoxia inducible factor 1 alpha (HIF-1*α*) expression and downstream vascular endothelial growth factor (VEGF) expression in neuroblastoma, which correlated with an antiangiogenic effect in tumor xenografts (Figure 1).^18^ Most recently, Lakoma *et al.* demonstrated that RG7388 also leads to decreased expression of HIF-1*α* and VEGF as well as the transcription to other pro-angiogenic factors in both p53 wild-type and p53 null-mutated cell lines. An *in vitro* angiogenesis assay confirmed these findings with a marked alteration and inhibition of angiogenesis with RG7388 treatment.^2^

The study by Lakoma *et al.* indeed verifies the potent efficacy of RG7388, which significantly upregulates p53 transcriptional targets and p53-mediated apoptosis in p53 wild-type neuroblastoma cell lines and orthotopic xenografts. Furthermore, RG7388 inhibits the p53-independent pathway of HIF-1*α*/VEGF, regardless of p53 status. Considering that nearly all neuroblastomas have an intact and wild-type;p53 in conjunction with the improved potency and bioavailability of this latest generation MDM2 inhibitor, RG7388 is a promising, non-genotoxic, targeted therapy for the treatment of neuroblastoma and other p53 wild-type solid tumors.

## Figures and Tables

**Figure 1:**
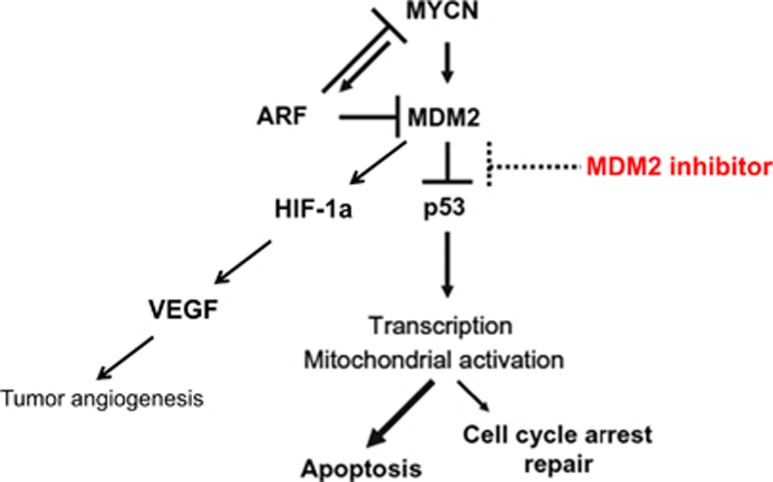
MDM2 inhibitors activate p53 through MDM2 binding. MDM2 is the primary negative regulator of p53, whereas ARF binds and inhibits MDM2 regulation of p53. ARF also blocks MYCN function yet is paradoxically activated by MYCN. By specifically binding to the p53-binding pocket of MDM2, MDM2 inhibitors (Nutlin-3a, RG7112 and RG7388) disconnect the upstream regulation of p53 by MDM2, which leads to stabilization of the p53 protein and higher intranuclear concentrations of p53. In a p53-independent manner, MDM2 inhibitors suppress HIF-1*α*, which is the major regulator of VEGF, leading to inhibition of tumor angiogenesis. In malignancies such as neuroblastoma, increased p53 activity leads to activation of apoptotic pathways rather than cellular repair pathways, suggesting a promising therapeutic option for p53 wild-type tumors.
